# Metagenomic Characterization and Molecular Screening of Pathogens in Freshwater Amphipods (*Gammarus lacustris*) from Kazakhstan: Implications for Aquaculture Biosecurity

**DOI:** 10.3390/pathogens15070663

**Published:** 2026-06-23

**Authors:** Marat Kumar, Symbat Suleimenova, Sardor Nuralibekov, Yermukhammet Kasymbekov, Temirlan Sabyrzhan, Kuanysh Isbekov, Saule Assylbekova, Victor Fefelov, Berik Pangereyev, Kobey Karamendin, Aidyn Kydyrmanov

**Affiliations:** 1 Laboratory for Ecology of Viruses, Research and Production Center for Microbiology and Virology, Almaty 050010, Kazakhstan; 2 Fisheries Research and Production Center, Almaty 050016, Kazakhstan; 3 Depertment of Biotechnology, Farabi University, Almaty 050040, Kazakhstan

**Keywords:** *Gammarus lacustris*, amphipods, metagenomics, freshwater virome, *Picornavirales*, *Dicistroviridae*, aquatic biosecurity, aquatic disease surveillance

## Abstract

Freshwater amphipods of the genus *Gammarus* are important trophic components of aquatic ecosystems and are increasingly considered a potential bioresource for aquaculture. However, their role in the maintenance and transmission of infectious agents remains poorly understood. This study evaluated the presence of major crustacean and fish pathogens in *Gammarus lacustris* populations from Kazakhstan and characterized associated viral communities using metagenomic sequencing. Six pooled samples collected from freshwater ecosystems across Kazakhstan were screened using PCR and RT-PCR assays targeting World Organisation for Animal Health (WOAH)-listed pathogens, including White Spot Syndrome Virus, Taura Syndrome Virus, Infectious Myonecrosis Virus, *Aphanomyces astaci*, and *Aphanomyces invadans*. In parallel, high-throughput sequencing (Illumina NovaSeq) was performed to assess virome composition and structure. No WOAH-listed pathogens were detected, suggesting a low detectable occurrence of major notifiable agents under the conditions of the present study. Metagenomic analysis revealed a virome dominated by RNA viruses, particularly picorna-like viruses (*Picornaviridae*), *Dicistroviridae*, and *Marnaviridae*. Phylogenetic and genome organization analyses identified potentially novel or highly divergent viral lineages within *Picornavirales*. Collectively, these findings suggest a favorable epizootiological profile of *G. lacustris* populations while highlighting freshwater amphipods as hosts of diverse and partially uncharacterized viral communities relevant to aquatic disease surveillance and aquaculture biosecurity.

## 1. Introduction

Aquatic invertebrates play a critical role in the epidemiology of infectious diseases affecting aquaculture species, acting as both reservoirs and vectors of pathogens transmitted through trophic pathways and aquatic ecological networks. The increasing intensification of aquaculture systems has amplified the risk of pathogen emergence and spread, with infectious diseases now recognized as a major constraint on global production and sustainability [1,2]. In this context, non-target organisms inhabiting aquatic ecosystems, including detritivorous invertebrates, represent an underappreciated component of pathogen transmission networks.

Among freshwater invertebrates, amphipods of the genus *Gammarus*, particularly *G. lacustris*, are widely distributed across the Holarctic region and dominate benthic communities in lakes, rivers, and wetlands [3]. Their ecological role as detritivores and scavengers places them at the intersection of multiple trophic levels, facilitating the accumulation of diverse microbial communities, including bacteria, fungi, and viruses [4,5]. Due to their high nutritional value, *Gammarus* spp. is increasingly utilized as live or processed feed in aquaculture, creating potential pathways for pathogen transfer to cultured fish and crustaceans.

Crustacean aquaculture is particularly vulnerable to viral pathogens, including White Spot Syndrome Virus (WSSV), Taura Syndrome Virus (TSV), and Infectious Myonecrosis Virus (IMNV), all of which are listed as notifiable diseases by the World Organisation for Animal Health (WOAH) [6]. These pathogens have caused extensive economic losses worldwide and continue to pose a major threat to aquaculture biosecurity [7]. In addition to viral agents, oomycete pathogens such as *Aphanomyces astaci* and *Aphanomyces invadans* are responsible for severe disease outbreaks in crustaceans and fish, further complicating disease management strategies.

Recent advances in high-throughput sequencing have revealed that aquatic organisms harbor highly diverse and largely unexplored viral communities [8]. Metagenomic approaches have become essential for identifying both known and previously undescribed viruses and for improving our understanding of viral evolution in aquatic ecosystems [9,10]. Studies of aquatic viromes have demonstrated that viral communities are highly dynamic and influenced by environmental conditions such as salinity, temperature [11], and host composition, often revealing previously unrecognized viral taxa and ecological interactions [12,13]. Given the recognized influence of salinity, temperature, and pH on aquatic viral community structure, these environmental variables were considered relevant for interpreting virome composition in the present study. In crustaceans, recent metagenomic investigations have identified extensive RNA virus diversity, including numerous novel viral lineages with unknown pathogenic potential [14]. These findings highlight the importance of virome-level analysis for assessing disease risks beyond traditional targeted diagnostics.

Despite these advances, the virome composition and pathogen burden of freshwater amphipods remain poorly characterized, particularly in Central Asia. This region is of growing importance for aquaculture development and biodiversity conservation, yet baseline data on pathogen prevalence and viral diversity in native invertebrate populations are largely lacking. This knowledge gap is particularly relevant in Kazakhstan, where the use of natural aquatic bioresources such as *G. lacustris* is increasing, while baseline information on pathogen diversity remains limited.

From a biosecurity perspective, aquatic invertebrates such as *Gammarus* may contribute to pathogen transmission through environmental exposure and trophic interactions. As detritivorous organisms inhabiting benthic habitats, they are continuously exposed to microorganisms present in sediments and surrounding water, potentially accumulating viral and microbial diversity. Integrating targeted molecular diagnostics with metagenomic virome analysis therefore provides a useful framework for evaluating both known pathogens and broader viral diversity relevant to aquaculture systems.

The objectives of this study were to screen *G. lacustris* populations in Kazakhstan for major crustacean and fish pathogens using molecular diagnostics, characterize associated viral communities through metagenomic sequencing, and evaluate their potential role in pathogen transmission, with implications for aquaculture biosecurity and the safe use of natural feed resources.

## 2. Materials and Methods

### 2.1. Sample Collection

**Figure 1:**
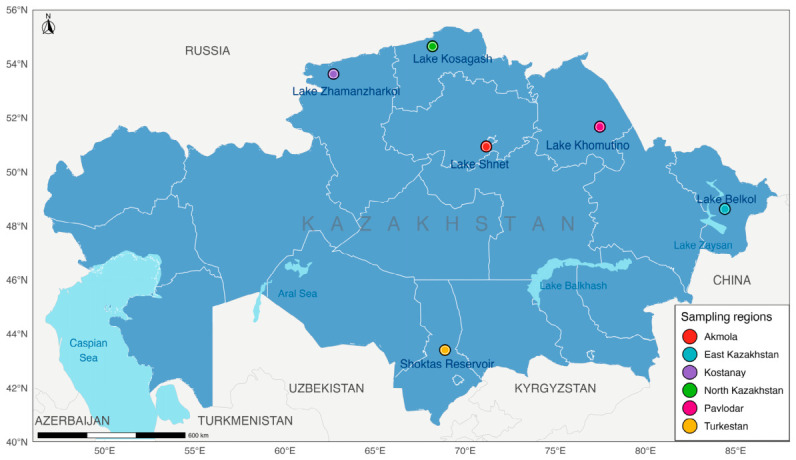
Geographic distribution of *G. lacustris* sampling sites across six freshwater ecosystems in Kazakhstan, including Lake Shnet (Akmola), Lake Belkol (East Kazakhstan), Lake Zhamanzharkol (Kostanay), Lake Khomutino (Pavlodar), Lake Kosagash (North Kazakhstan), and Shoktas Reservoir (Turkestan).

A total of six pooled biomass samples of *G. lacustris* were collected from freshwater bodies across six regions of Kazakhstan—Lake Shnet, Akmola Region (AKM); Lake Belkol, East Kazakhstan Region (EKZ); Lake Zhamanzharkol, Kostanay Region (KST); Lake Khomutino, Pavlodar Region (PVL); Lake Kosagash, North Kazakhstan Region (NKZ); Shoktas Reservoir, Turkestan Region (TRK)—between June and September 2025 (Figure 1). Six composite samples were generated, each comprising five pooled sites per reservoir. To ensure representative sampling and account for spatial heterogeneity within each reservoir, *Gammarus* specimens were collected from five independent sampling sites at each location. Sampling was conducted in the littoral zone at depths of approximately 0.3–1.5 m, where amphipod populations are typically most abundant. Biomass was collected using a fine-mesh plankton net (150 μm mesh size) and rinsed with ambient lake water to remove debris and sediment particles. Approximately 40–50 g of biomass was obtained from each site, and the material was combined to produce a single composite sample weighing 180–200 g per reservoir. Samples were transported to the laboratory in containers with ambient water and stored at −80 °C until further molecular screening and metagenomic analyses.

### 2.2. Environmental Parameter Measurement

Environmental parameters, including pH, salinity (mg·dm^−3^), and temperature (°C), were measured at each sampling site. Water salinity in the reservoirs was measured using a PAL-SALT (model 4250, Atago, Tokyo, Japan). These variables were used to characterise environmental gradients among freshwater ecosystems inhabited by *Gammarus* populations. Descriptive statistics and graphical visualisation were used to evaluate variability across sampling sites.

### 2.3. Viral Nucleic Acid Extraction, Library Preparation, and High-Throughput Sequencing

From each regional composite sample, a representative subsample of 10–15 *Gammarus* individuals (approximately 50–100 mg wet biomass per individual) was selected for downstream nucleic acid extraction. Selected specimens were transferred into 2 mL microcentrifuge tubes containing sterile phosphate-buffered saline (PBS, pH 7.4) and stainless-steel beads for homogenization. Samples were homogenised using a TissueLyser (Qiagen, Hilden, Germany) at 25 Hz for 3 min, followed by centrifugation (Eppendorf, Hamburg, Germany) at 4500× *g* for 10 min at 4 °C.

The resulting supernatants were collected and filtered through 0.45 μm syringe filters to remove cellular debris and larger particles. Viral nucleic acids were extracted from filtered supernatants using the QIAamp Viral RNA Mini Kit (Qiagen, Hilden, Germany) according to the manufacturer’s instructions. Double-stranded complementary DNA (cDNA) was synthesized from extracted RNA using the QIAseq RNA Library Kit (Qiagen, Hilden, Germany) prior to downstream library preparation. No targeted enrichment strategy was applied to selectively separate RNA and DNA viruses; therefore, both RNA-derived and DNA viral sequences present in the extracted nucleic acid pool were retained for untargeted metagenomic sequencing.

Metagenomic libraries were prepared using the TruSeq Nano DNA Library Preparation Kit (Illumina, San Diego, CA, USA) with an average insert size of approximately 350 bp. Library quality and fragment size distribution were assessed using an Agilent 2100 Bioanalyzer (Agilent Technologies, Santa Clara, CA, USA), and concentrations were quantified using a Qubit fluorometer (Thermo Fisher Scientific, Waltham, MA, USA). Sequencing was performed by Macrogen Inc. (Daejeon, Republic of Korea) on the Illumina NovaSeq X Plus platform (Illumina, San Diego, CA, USA) (150 bp PE), yielding approximately 70–90 million reads per sample.

Environmental water controls and laboratory blank controls were not included in the present study due to logistical constraints. To reduce the influence of potential contaminant signals, downstream analyses focused primarily on dominant viral taxa supported by higher contig abundance and longer genomic regions.

### 2.4. Viral Identification and Annotation

Data processing was performed on a local Linux-based computational system. Raw paired-end metagenomic reads were processed using an automated workflow implemented in LazyPipe (v3.1) [15]. Initial quality control and adapter trimming were performed using fastp (v0.23.2) [16] with the following parameters: -q 15 -u 40 -l 30.

Because no complete reference genome suitable for host filtering is currently available for *G. lacustris*, reliable host-genome depletion could not be fully achieved. Therefore, quality-filtered reads were mapped to the human reference genome (GCF_000001405.40) using BWA (v0.7.17) [17] to reduce potential contamination arising during sample processing. Reads aligning to the human genome were removed with Sambamba (v0.8.2) [18], and the remaining reads were retained for downstream analyses. De novo assembly was performed using MEGAHIT (v1.2.9) [19] with the “meta-sensitive” preset. Contigs shorter than 200 bp were excluded from further analysis.

Taxonomic annotation followed a two-step similarity search strategy. First, assembled contigs were screened against a viral nucleotide database using Minimap2 (v2.24) [20] to identify candidate viral sequences. Candidate hits were subsequently validated using BLASTN (BLAST+ v2.13.0) against the NCBI viral nucleotide database (downloaded January 2025) with an E-value threshold of 1 × 10^−5^**.** Only high-confidence viral hits with significant similarity to reference viral sequences were retained for downstream analyses and inclusion in Supplementary Table S1.

### 2.5. Data Analysis

Data analysis and visualization were performed in RStudio (v2026.01.1) using R (v4.6.0). Bar plots were generated using the ggplot2 package (v4.0.3; https://ggplot2.tidyverse.org/, accessed on 10 March 2026), and heatmaps were constructed using the ComplexHeatmap package (v2.22.0; https://jokergoo.github.io/ComplexHeatmap-reference/book/, accessed on 10 March 2026). Hierarchical clustering was performed using Euclidean distance and complete linkage. To improve comparability among taxa with differing abundance levels, read counts were log_10_-transformed [log_10_(x + 1)] prior to heatmap visualization. 

### 2.6. Phylogenetic Analysis

For high-abundance viral taxa (e.g., picorna-like and dicistrovirus-like viruses), the longest assembled contigs were selected for phylogenetic and genome organization analyses. Reference sequences were selected based on the closest homologs identified through BLASTn and BLASTx searches together with representative members of the corresponding viral taxa. For dominant picorna-like and dicistrovirus-like viruses, quality-filtered metagenomic reads were additionally mapped to the closest reference genomes identified through BLASTn analysis using BWA (v0.7.17) [17]. Alignment files were processed using SAMtools (v1.20), and consensus sequences were reconstructed from mapped reads. Genome coverage and sequencing depth were calculated, and sequence variation relative to reference genomes was assessed through variant calling using BCFtools (v1.20) for comparative analysis among sampling sites. Multiple sequence alignments were generated using ClustalW implemented in MEGA11 (v11.0.13; https://www.megasoftware.net/, accessed on 27 April 2025), manually inspected, and trimmed prior to phylogenetic reconstruction using the maximum likelihood (ML) method with 1000 bootstrap replicates. Conserved protein domains and amino acid homology, including RNA-dependent RNA polymerase (RdRp) and structural polyprotein regions, were evaluated for selected viral sequences. Putative open reading frames (ORFs) were predicted using Geneious Prime (v2026.0.2) to identify coding regions within assembled viral contigs.

### 2.7. Molecular Screening of Crustacean Pathogens

PCR and RT-PCR assays were performed to screen for major crustacean pathogens, including WSSV, TSV, IMNV, and oomycete fungi *Aphanomyces astaci* and *Aphanomyces invadans* using previously published primers and protocols [21]. Sterile nuclease-free water was included as a negative control in each PCR run to monitor contamination. Amplified products were visualized by agarose gel electrophoresis.

### 2.8. Use of Generative Artificial Intelligence (GenAI)

The authors used generative artificial intelligence (GenAI) tools, including ChatGPT (GPT-5.5, OpenAI, San Francisco, CA, USA) and Perplexity AI (Perplexity AI Inc., San Francisco, CA, USA), to assist with language editing, text refinement, and improvement of manuscript clarity and structure. All scientific content, including study design, data collection, analysis, interpretation, and conclusions, was developed, reviewed, and verified solely by the authors, who take full responsibility for the accuracy, integrity, and originality of the work.

## 3. Results

### 3.1. Sequencing Output and Data Quality

Metagenomic sequencing of *Gammarus* samples yielded between 71.59 and 89.54 million raw paired-end reads per sample (Table 1). Following quality control (QC) filtering, 68.26–86.14 million reads per sample were retained, corresponding to 95.35–97.22% of the raw sequencing data.

After contamination-related filtering, the number of reads retained for downstream analysis varied considerably among sampling sites. Samples from Lake Shnet (Akmola Region) and Lake Belkol (East Kazakhstan Region) exhibited the highest proportions of retained reads (95.80% and 95.22%, respectively), indicating a lower proportion of sequences removed during filtering. In contrast, the Pavlodar sample (Lake Khomutino) showed a markedly lower proportion of retained reads (21.64%), suggesting a higher proportion of non-target sequences in the raw dataset.

### 3.2. Environmental Characteristics and Their Relationship to Viral Richness

The physicochemical parameters of the six freshwater bodies sampled in this study are summarized in Table 2. Environmental conditions varied across sampling sites, with water temperature ranging from 14.6 °C in Lake Zhamanzharkol to 23.1 °C in Lake Khomutino. Salinity also differed substantially, ranging from 281 mg·dm^−3^ in Lake Belkol to 2286 mg·dm^−3^ in Lake Kosagash. In contrast, pH values showed relatively limited variation (7.19–8.10), indicating generally neutral to slightly alkaline conditions across freshwater ecosystems.

**Figure 2:**
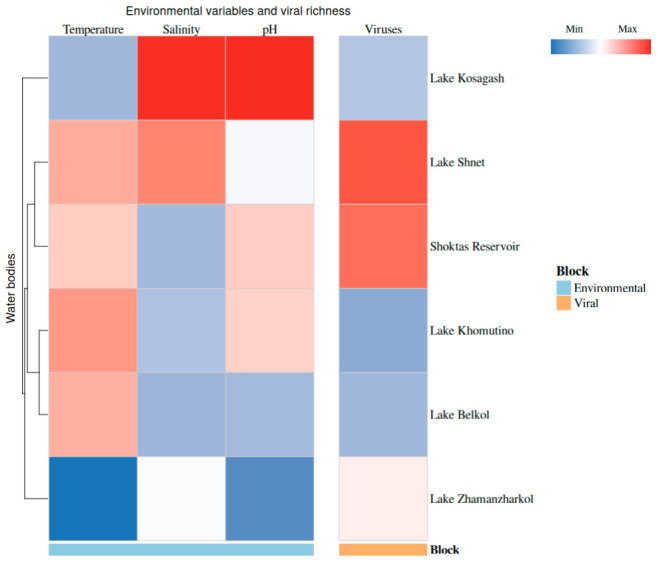
Relationship between environmental variables (temperature, salinity, and pH) and viral species richness in *Gammarus* populations across freshwater ecosystems in Kazakhstan. Color gradients represent normalized values ranging from low (blue) to high (red). Hierarchical clustering illustrates similarities among sampling sites based on environmental conditions and associated viral richness.

To explore potential relationships between environmental conditions and virome structure, viral richness was evaluated in relation to measured physicochemical variables (Figure 2). Variation in viral richness appeared to coincide with differences in temperature and salinity among sites.

Lake Shnet (Akmola Region) and Shoktas Reservoir (Turkestan Region) exhibited higher viral richness, whereas Lake Kosagash (North Kazakhstan Region) and Lake Khomutino (Pavlodar Region) showed lower richness despite contrasting physicochemical characteristics. For example, Lake Kosagash combined high salinity with relatively low viral richness, whereas Lake Khomutino exhibited the highest recorded temperature but lower viral diversity. These observations suggest that multiple environmental and ecological factors may contribute to shaping virome composition.

Spearman correlation analysis suggested a positive association between viral richness and water temperature (ρ = 0.71, *p* < 0.05) and a moderate association with salinity (ρ = 0.58, *p* < 0.05), whereas no significant relationship was observed with pH (ρ = 0.21, *p* > 0.05). However, given the limited number of sampling sites, these findings should be interpreted cautiously and viewed as indicative of potential environmental trends rather than definitive ecological relationships.

Heatmap visualization and hierarchical clustering further illustrated differences among sampling sites. Lake Shnet and Shoktas Reservoir formed a cluster associated with higher viral richness, whereas Lake Khomutino (Pavlodar Region) and Lake Belkol (East Kazakhstan Region) grouped separately and showed lower-to-intermediate viral richness. Although some clustering patterns coincided with environmental similarities, the observed variation suggests that no single physicochemical factor alone appeared to explain virome composition. Overall, these findings suggest that environmental gradients may contribute to shaping viral diversity associated with freshwater *G. lacustris* populations, although broader sampling will be necessary to clarify these relationships.

### 3.3. Virome Composition Across Sampling Sites

**Figure 3:**
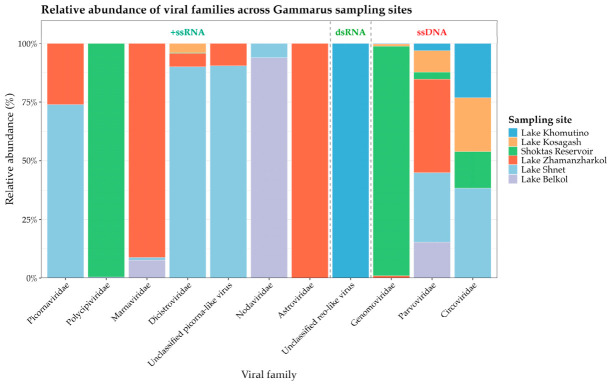
Relative abundance of viral families detected in *G. lacustris* across six freshwater ecosystems in Kazakhstan. Viral families are grouped by genome type (+ssRNA, dsRNA, ssDNA). Bar plots show the proportional contribution of each sampling site to the total abundance of each viral family.

Metagenomic analysis revealed a diverse viral community associated with *G. lacustris*, comprising multiple RNA and DNA virus families (Figure 3). Overall, the virome was dominated by positive-sense single-stranded RNA (+ssRNA) viruses, detected at all sampling sites.

Among +ssRNA viruses, members of the families *Picornaviridae*, *Dicistroviridae*, and *Marnaviridae* were particularly abundant, although their relative contributions varied markedly between locations. For instance, *Dicistroviridae* and unclassified picorna-like viruses were highly represented in Lake Shnet and Lake Khomutino, whereas *Marnaviridae* dominated in Lake Zhamanzharkol. By contrast, Lake Kosagash was characterised by a strong predominance of *Polycipiviridae*.

Double-stranded RNA (dsRNA) viruses were primarily represented by unclassified reo-like viruses, detected almost exclusively in Lake Khomutino, indicating site-specific viral assemblages. In contrast, Single-stranded DNA (ssDNA) viruses, including members of the families *Parvoviridae*, *Circoviridae*, and *Genomoviridae*, were also detected across multiple sites. *Genomoviridae* showed a striking dominance in Shoktas Reservoir, while *Parvoviridae* and *Circoviridae* exhibited more heterogeneous distributions, with notable contributions in Lake Zhamanzharkol, Lake Belkol, and Lake Shnet.

### 3.4. Viral Species Composition and Host Association

**Figure 4:**
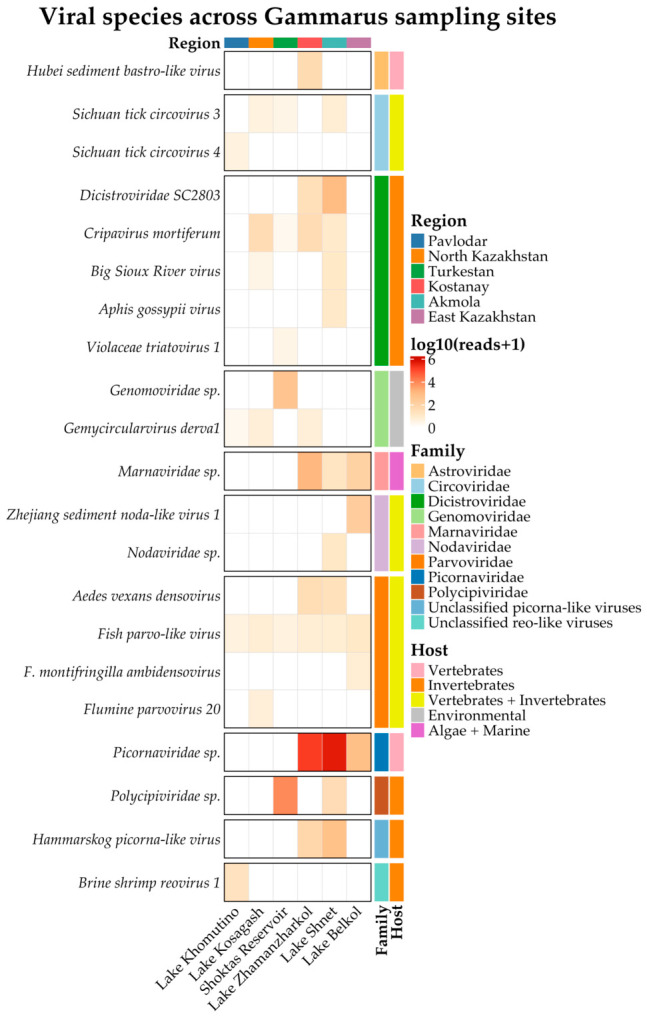
Heatmap of viral species composition associated with *G. lacustris* across six freshwater ecosystems in Kazakhstan. Rows represent viral species and columns correspond to sampling sites. Color intensity indicates relative abundance based on log10-transformed read counts. Annotation bars indicate sampling region, viral family classification, and predicted host association.

At the species level, a broad diversity of viral taxa was identified across the six sampling sites, including viruses associated with invertebrates, vertebrates, and aquatic environmental reservoirs (Figure 4). Both RNA and DNA viruses were detected, indicating that the metagenomic profiles associated with *Gammarus lacustris* reflected a mixture of host-related and environmentally derived viral sequences.

Because viral metagenomic assemblies are frequently fragmented, interpretation focused mainly on dominant taxa supported by higher contig abundance, repeated independent detections, and longer assembled regions. This approach was used to reduce overinterpretation of assignments based on short or low-confidence contigs [15,22].

Across the sampled ecosystems, members of the families *Picornaviridae* and *Dicistroviridae* were the dominant viral groups, although their abundance differed considerably between locations. The highest number of picorna-like viral contigs was observed in the Akmola sample (Lake Shnet), where 391 contigs were identified (Supplementary Table S1). These sequences ranged from 200 to 9520 bp and showed nucleotide identities between 78.65% and 99.29%, pointing to the presence of both closely related and more divergent picorna-like lineages. A similar pattern was observed in the Kostanay region (Lake Zhamanzharkol), where 241 picorna-like contigs were recovered, with sequence identities ranging from 82.6% to 99.2%.

Dicistrovirus-related sequences were also common. In the Akmola sample, 24 contigs were assigned to *Dicistroviridae* SC2803, ranging from 217 to 2469 bp and showing nucleotide identities of 91.06–96.66%. Sequences related to *Cripavirus mortiferum* and *Big Sioux River virus* were detected in the same sample. Similar dicistrovirus-related contigs were observed in the Kostanay sample, with nucleotide identity values ranging from 91.95% to 94.96%.

Part of the detected viral diversity likely reflects interactions between *G. lacustris* and surrounding aquatic microbial communities. Viruses associated with algae, protists, and environmental reservoirs were identified at several sites. Members of the family *Marnaviridae*, commonly reported from aquatic microbial systems, showed sequence identities ranging from 77.23% to 99.57%, suggesting the presence of both conserved and divergent lineages. Sediment-associated viruses, including *Hubei sediment bastro-like virus* and *Zhejiang sediment noda-like virus 1*, were also identified, further indicating environmental inputs into the observed viral communities.

DNA viruses were present at all sampling sites, although generally at lower abundance than RNA viruses. Members of the family *Parvoviridae* were among the most widespread. *Aedes vexans densovirus* was detected at several locations with sequence identities approaching 100%, whereas fish-associated parvo-like viruses appeared in multiple regions. These findings most likely reflect environmental exposure or indirect ecological associations rather than confirmed host infection.

Several low-abundance viral taxa, including circoviruses and genomoviruses, were also observed. Sequences related to *Sichuan tick circovirus* and *Gemycircularvirus derva1* showed high similarity to previously reported environmental viruses. In addition, *Brine shrimp reovirus 1* was detected only in Lake Khomutino, indicating local differences in viral composition among freshwater ecosystems.

Viruses associated with different host groups frequently co-occurred within the same samples. Although invertebrate-associated viruses predominated, vertebrate- and environment-associated viruses were also consistently present. Considering the ecology of *G. lacustris*, including detrital feeding and frequent contact with sediment, some viral sequences may reflect dietary material, environmental exposure, or indirect ecological interactions rather than active host infection.

Overall, viral composition differed substantially among sampling sites, with each freshwater ecosystem showing a distinct combination of dominant taxa. These differences likely reflect variation in local ecological conditions and habitat characteristics. No WOAH-listed pathogens were identified under the conditions of this study; however, the results should be interpreted cautiously because of pooled sampling, fragmented assemblies, and variation in sequencing depth. Continued surveillance and comparative molecular characterization are needed to better understand the diversity and ecological relevance of viruses associated with freshwater amphipods.

### 3.5. Genetic Variation of Dominant Viruses

Picorna-like viruses represented the dominant viral group detected in the dataset and were selected for additional genetic characterization. To compare sequence variation among sampling sites, reference-based mapping and consensus genome reconstruction were performed using dominant picorna-like viral datasets.

The highest abundance of picorna-like viral contigs was detected in the Akmola (Lake Shnet) and Kostanay (Lake Zhamanzharkol) samples, while lower abundance was observed in East Kazakhstan (Lake Belkol). Reads from the Akmola and Kostanay datasets were mapped against *Picornaviridae* sp. ON162258.1, which showed the highest similarity in BLASTn searches. Near-complete consensus genomes of 8340 nt (AKM) and 8341 nt (KST) were reconstructed, with genome coverage of 97.93% and 97.63%, respectively. Mean sequencing depth reached 16,052× in the Akmola sample and 5176× in the Kostanay sample (Supplementary Table S2).

Variant analysis identified 538 nucleotide substitutions in the Akmola consensus sequence and 476 substitutions in the Kostanay sequence relative to *Picornaviridae* sp. ON162258.1. Although both viruses clustered within the same reference lineage, the observed differences indicate sequence divergence between dominant picorna-like viruses detected in different freshwater ecosystems.

The East Kazakhstan picorna-like virus showed greater similarity to the *StochSRVP3 picorna-like virus* (OQ722329.1) and was therefore analyzed separately. Reference-based mapping generated an 8576 nt consensus sequence containing nine nucleotide substitutions relative to the closest reference genome.

**Figure 5:**
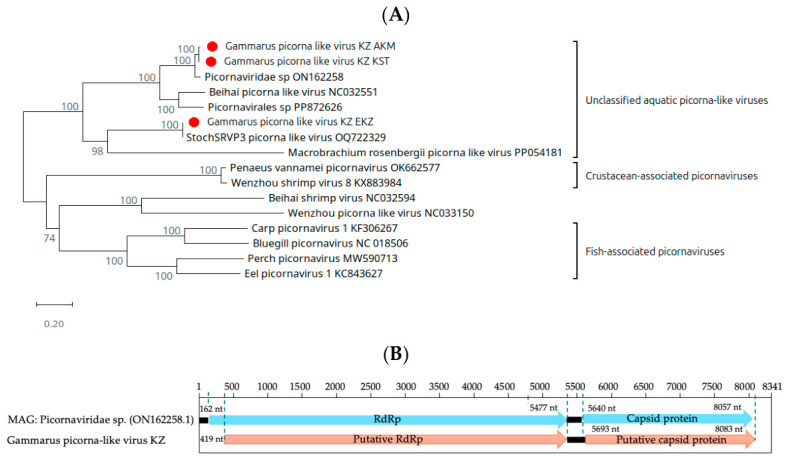
Phylogenetic analysis and genome organization of dominant picorna-like viruses detected in *G. lacustris*. (**A**) Maximum-likelihood phylogenetic tree of consensus sequences from freshwater ecosystems in Kazakhstan. Kazakhstan *Gammarus*-associated sequences are highlighted in red. Bootstrap support values (>60%) are shown at nodes. The scale bar represents substitutions per site. (**B**) Genome organization of the dominant picorna-like viral contig recovered from the Akmola sample, showing putative RNA-dependent RNA polymerase (RdRp) and capsid protein regions.

Phylogenetic analysis of the reconstructed consensus sequences (AKM, 8340 nt; KST, 8341 nt; EKZ, 8576 nt) placed the Kazakhstan *Gammarus*-associated viruses within a clade of unclassified aquatic picorna-like viruses (Figure 5A). The Akmola and Kostanay sequences clustered closely with *Picornaviridae* sp. ON162258.1, whereas the East Kazakhstan sequence grouped with *StochSRVP3 picorna-like virus* (OQ722329.1), indicating regional genetic variation among dominant picorna-like viruses.

Genome organization analysis of the longest assembled contig recovered from the Akmola sample (8297 nt) revealed a conserved picorna-like architecture containing putative RNA-dependent RNA polymerase (RdRp) and capsid protein regions (Figure 5B). BLASTn comparison showed 89.72% nucleotide identity to *Picornaviridae* sp. ON162258.1, while amino acid similarity in the RdRp region reached 94.08%.

Dicistrovirus-like sequences were detected in both the Akmola and Kostanay samples and represented one of the dominant RNA viral groups in the dataset. In the Akmola sample, 24 contigs related to *Dicistroviridae* SC2803 were identified, ranging from 217 to 2469 bp and showing 91.06–96.66% nucleotide identity. Sequences related to *Cripavirus mortiferum* and *Big Sioux River virus* were also detected. Similar dicistrovirus-related contigs were observed in the Kostanay sample, with nucleotide identity values ranging from 91.95% to 94.96%.

**Figure 6:**
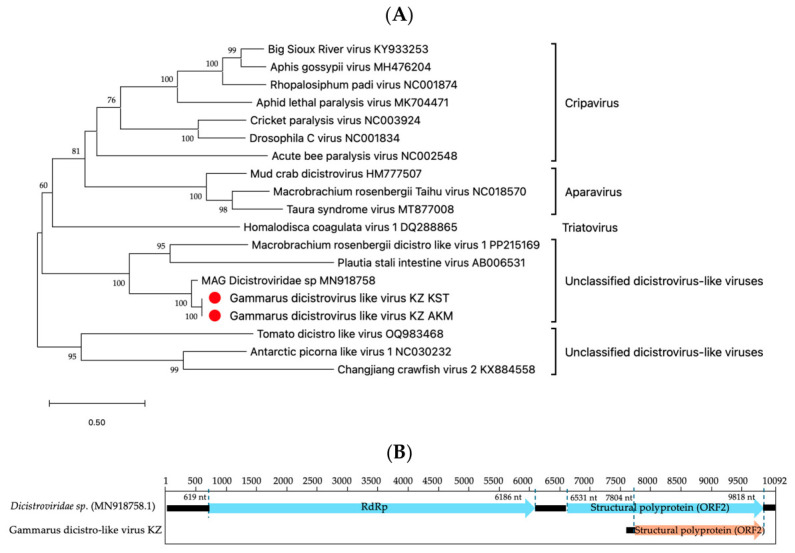
Phylogenetic analysis and genome organization of dicistrovirus-like viruses detected in *G. lacustris*. (**A**) Maximum-likelihood phylogenetic tree of dicistrovirus-like structural polyprotein sequences detected in Kazakhstan freshwater ecosystems. Kazakhstan *Gammarus*-associated sequences are highlighted in red. Bootstrap values (>60%) are shown at branch nodes. The scale bar indicates substitutions per site. Genus-level groups are indicated where applicable. (**B**) Genome organization of the representative dicistrovirus-like contig recovered from the Akmola sample, showing the structural polyprotein region (ORF2) in comparison with the closest reference sequence (*Dicistroviridae* sp. MN918758.1).

Phylogenetic analysis placed the Kazakhstan *Gammarus*-associated sequences within a clade of unclassified dicistrovirus-like viruses (Figure 6A). The Akmola (AKM; 2469 bp) and Kostanay (KST; 2033 bp) sequences clustered closely together with strong bootstrap support (100) and formed a sister lineage to *Dicistroviridae* sp. MN918758.1. This clustering supports the presence of closely related dicistrovirus-like lineages in geographically separated freshwater ecosystems.

Genome organization of the representative Akmola contig revealed a structure consistent with dicistrovirus-like viruses, including a structural polyprotein region (ORF2) (Figure 6B). BLASTn comparison showed 93.15% nucleotide identity to *Dicistroviridae* sp. MN918758.1, while protein-level analysis indicated approximately 95% amino acid identity to previously described dicistrovirus structural proteins. The assembled contig (2469 bp) contained a near-complete ORF2 coding region (671 amino acids), and its genomic organization was comparable to that of the reference genome. Although only a partial genome was recovered, the available evidence did not support a definitive genus-level assignment. Together, these findings support the presence of related dicistrovirus-like lineages associated with *G. lacustris* populations in Kazakhstan.

## 4. Discussion

This study provides the first comprehensive characterization of pathogen occurrence and viral diversity in *G. lacustris* populations across multiple freshwater ecosystems in Kazakhstan. By integrating targeted molecular screening with viral metagenomics, the study offers new insights into both the biosecurity status of freshwater amphipod populations and the ecological complexity of amphipod-associated viromes. The results revealed a diverse assemblage of viral taxa, with no detectable evidence of several economically important aquatic pathogens.

The absence of major WOAH-listed pathogens, including WSSV, TSV, IMNV, and *Aphanomyces* spp., suggests a favorable epizootiological status of the studied G. lacustris populations. These pathogens are responsible for substantial economic losses in global aquaculture and represent major threats to aquatic animal health [1,7,21]. Accordingly, the findings do not support the role of *G. lacustris* populations in Kazakhstan as major reservoirs of these high-risk pathogens. Nevertheless, negative PCR and metagenomic findings should be interpreted cautiously, as molecular detection is influenced by pathogen abundance, assay sensitivity, sequencing depth, nucleic acid integrity, and sampling design. Low-abundance, transient, fragmented, or latent infections may therefore remain undetected, particularly in pooled environmental samples. Recent advances in metatranscriptomic approaches have substantially improved sensitivity for detecting emerging and low-prevalence pathogens [2], highlighting the value of integrating high-throughput sequencing into aquatic disease surveillance frameworks. Consequently, the present findings should be interpreted as evidence of low detectable pathogen occurrence under the conditions of this study rather than confirmed pathogen absence.

Environmental variability likely played an important role in shaping virome composition across sampling locations. Salinity and temperature emerged as important environmental factors associated with variation in viral richness, consistent with previous studies identifying physicochemical gradients as major drivers of aquatic viral community structure [11,23,24,25,26,27]. However, the observed relationships were not consistent across all sampling sites, suggesting that multiple ecological and environmental factors likely influence virome structure in freshwater ecosystems. Similar spatial heterogeneity in aquatic viral communities has been reported in previous ecological studies, where ecological conditions, host availability, and habitat characteristics contribute to variation in viral diversity [23,24,25,26,27]. Given the limited number of sampling sites included in the present study, these environmental associations should be interpreted cautiously and considered indicative of broader ecological patterns rather than definitive causal relationships.

The ecological characteristics of *G. lacustris* likely contribute to the diversity of detected viral communities. As a benthic detritivore inhabiting littoral and sediment-associated habitats, Gammarus continuously interacts with microbial biofilms, algae, protists, decomposing organic matter, and sediment-associated microorganisms [28]. Such ecological interactions increase exposure to viruses originating from diverse surrounding sources and likely explain the mixed viral signatures observed in the present study. Amphipods are also recognized as hosts for diverse parasite assemblages and microbial communities, reflecting frequent interactions with microorganisms and pathogens [5,29,30,31]. Consequently, the virome detected in *G. lacustris* most likely represents a combination of host-associated viruses and environmentally acquired viral signatures rather than a strictly host-specific viral assemblage.

Within this ecological context, RNA viruses clearly dominated the amphipod virome, particularly picorna-like viruses and members of the family *Dicistroviridae*, a pattern commonly observed in invertebrate-associated viromes [8,9,11,14]. *Picornaviridae*-related sequences represented the most abundant viral group, especially in the Akmola and Kostanay samples, where 391 and 241 independent contigs were recovered, respectively. The high abundance and repeated detection of picorna-like viral contigs across independent samples suggest that these sequences likely represent biologically relevant components of the detected virome rather than isolated assembly artefacts or sporadic low-level signals.

The broad range of sequence identities and observed genetic variation indicates the coexistence of both conserved and divergent picorna-like lineages within freshwater ecosystems. Recovery of long genomic regions from dominant picorna-like viruses further supports the presence of genetically distinct RNA viral lineages. In addition, reference-based mapping and consensus genome reconstruction revealed substantial sequence variation between geographically separated freshwater ecosystems, supporting the circulation of genetically related but distinct viral lineages in Kazakhstan freshwater habitats.

Although the pathogenic significance of these picorna-like viruses in *G. lacustris* remains unclear, their repeated detection across samples warrants further investigation, particularly because members of the order *Picornavirales* include viruses associated with disease in diverse aquatic organisms [14,32].

Polycipiviridae-related sequences were repeatedly detected, particularly in the Turkestan sample, where multiple independent contigs, including long genomic fragments (up to 3299 bp), matched previously reported environmental *Polycipiviridae* genomes recovered from sediment-associated metagenomes. Members of this family are increasingly reported from arthropod-associated and environmental viromes, although their host range and biological significance remain poorly understood [9,33]. Their repeated detection further highlights the diversity of RNA viruses present in freshwater ecosystems.

Several additional viral taxa detected in the present study may have ecological and biosecurity relevance. Members of the family *Dicistroviridae*, including *Dicistroviridae* SC2803 identified in the Akmola sample, occurred at moderate abundance and are noteworthy because dicistroviruses are recognized arthropod-associated RNA viruses, some of which have been reported in economically important insects and crustaceans [34,35,36,37]. Nodavirus-related sequences, represented by *Zhejiang sediment noda-like virus 1*, were also detected at lower abundance. Although no major WOAH-listed crustacean pathogens were identified, the detection of nodavirus-related sequences remains notable because members of the family *Nodaviridae* have been linked to disease in fish and aquatic invertebrates [13,14]. Similarly, sporadic viral signatures such as *Brine shrimp reovirus 1* and fish parvo-like virus may reflect localized environmental exposure and interactions between amphipods and surrounding aquatic reservoirs.

Several viral assignments, including Aedes vexans densovirus and *Gemycircularvirus derva1*, exhibited near-complete (99–100%) nucleotide similarity to previously described reference genomes, increasing confidence in classification and suggesting that these viral signatures likely reflect genuine biological or environmental occurrence within sampled ecosystems. Nevertheless, metagenomic interpretation of virome composition requires careful consideration, particularly in aquatic systems where trophic interactions, environmental inputs, and contamination may complicate ecological inference and host–virus assignment [2,10,13,14]. Although the present study prioritized dominant viral taxa supported by higher contig abundance, multiple independent contigs, and longer genomic regions, metagenomic detection alone does not confirm active replication, pathogenicity, or host specificity in *G. lacustris*. Likewise, phylogenetic analyses were based primarily on conserved coding regions, including RdRp, and should therefore be considered supportive of evolutionary placement rather than definitive genus- or species-level classification. Additional targeted sequencing, complete genome recovery, and confirmatory molecular analyses will be necessary to clarify host associations, biological significance, and formal taxonomic placement of these viral taxa.

In addition to host-associated viral groups, part of the detected virome likely reflects surrounding environmental and microbial inputs. Members of the family *Marnaviridae*, commonly associated with algae and protists, were detected across multiple sampling sites, suggesting environmental contributions to virome composition. Likewise, sediment-associated viruses, including *Hubei sediment bastro-like virus*, further support the influence of benthic habitats on viral diversity [13,38].

DNA viruses occurred at lower abundance, with members of the family *Parvoviridae* representing the predominant DNA-associated taxa [39]. This pattern is consistent with previous aquatic invertebrate virome studies, where RNA viruses generally predominate [11,14]. Low-abundance vertebrate-associated viral signatures may reflect environmental exposure or trophic interactions rather than confirmed infection of amphipod hosts, highlighting the need for cautious interpretation of metagenomic host assignment [10,13,14].

The occurrence of site-specific viral signatures, including the exclusive presence of certain viruses in individual lakes, suggests that habitat-related ecological factors contribute to variation in virome composition. Spatial heterogeneity is a common feature of aquatic viral communities and likely reflects interactions among habitat characteristics, microbial diversity, host ecology, and ecosystem connectivity [23,40]. Together, these findings suggest that *G. lacustris* may serve as an ecological interface linking host-associated and environmentally acquired viral communities, with virome composition shaped by both habitat-mediated transmission and biological interactions.

Notably, the virome composition observed in G. lacustris resembles recent metagenomic findings from Artemia cysts collected from saline lakes in Kazakhstan, where picorna-like and dicistrovirus-related sequences similarly predominated [41]. The recurrence of related RNA viral groups across different crustacean hosts and aquatic environments may reflect a broader RNA virosphere associated with freshwater and saline invertebrate communities.

From an applied perspective, the absence of major notifiable pathogens under the conditions of this study may support the potential suitability of *G. lacustris* as a candidate feed resource for aquaculture. Nevertheless, the substantial diversity of detected viral sequences, including viruses with uncertain host range and pathogenic potential, highlights the importance of continued molecular surveillance and ecological risk assessment. Overall, the present study expands current understanding of freshwater amphipod-associated viromes and demonstrates the value of integrating metagenomic approaches into aquatic biosecurity research. The detection of divergent viral lineages further emphasizes the importance of aquatic invertebrates as reservoirs of previously unexplored viral diversity and provides a foundation for future investigations into freshwater viral ecology.

## Figures and Tables

**Table 1:** Sequencing summary of six pooled *G. lacustris* samples collected from freshwater ecosystems in Kazakhstan, including total raw reads, QC-passed reads, and reads retained after contamination filtering.

Sample ID	Region (Basin)	Water Body	Total Reads	QC-Passed Reads (%)	Retained Reads (%)
*Gammarus* PVL	Pavlodar	Lake Khomutino	75,980,166	73,718,702 (97.03%)	16,443,138 (21.64%)
*Gammarus* NKZ	North Kazakhstan	Lake Kosagash	82,912,446	79,542,260 (95.94%)	69,627,034 (83.97%)
*Gammarus* TRK	Turkestan	Shoktas Reservoir	71,585,656	68,255,682 (95.35%)	67,979,762 (94.90%)
*Gammarus* KST	Kostanay	Lake Zhaman-zharkol	77,524,492	75,366,650 (97.22%)	50,721,136 (65.42%)
*Gammarus* AKM	Akmola	Lake Shnet	89,545,352	86,135,526 (96.19%)	85,877,502 (95.80%)
*Gammarus* EKZ	East Kazakhstan	Lake Belkol	79,030,976	75,958,512 (96.11%)	75,248,514 (95.22%)

**Table 2:** Physicochemical parameters of freshwater bodies sampled for *Gammarus* populations across different regions of Kazakhstan.

Region	Water Body	pH	Salinity (mg·dm^−3^)	Temperature (°C)
Pavlodar	Lake Khomutino	7.74	401	23.1
North Kazakhstan	Lake Kosagash	8.1	2286	17.4
Turkestan	Shoktas Reservoir	7.75	312	21.7
Kostanay	Lake Zhamanzharkol	7.19	988	14.6
Akmola	Lake Shnet	7.6	1830	22.6
East Kazakhstan	Lake Belkol	7.37	281	22.5

## Data Availability

The metagenomic datasets generated during this study are publicly available in the NCBI BioProject database under accession number PRJNA1454535, with associated BioSample accessions SAMN57311258–SAMN57311263 and corresponding SRA records.

## Supplementary Materials

The following supporting information can be downloaded at https://www.mdpi.com/article/10.3390/pathogens15070663/s1, Table S1: Comprehensive list of viral taxa identified in *Gammarus lacustris* samples collected from six freshwater ecosystems across Kazakhstan; Table S2: Reference-based genetic variation analysis of dominant picorna-like viruses.
