# Supplementary material for: Metagenomic Characterization and Molecular Screening of Pathogens in Freshwater Amphipods (Gammarus lacustris) from Kazakhstan: Implications for Aquaculture Biosecurity
Source: Pathogens. 2026 Jun 23;15(7):663. doi: 10.3390/pathogens15070663 (PMC13415222; doi:10.3390/pathogens15070663)
Supplement: Supplementary file 1 [file pathogens-15-00663-s001.zip › pathogens-4294067-supplementary.pdf]

Supplementary Information

Metagenomic Characterization and Molecular Screening of Pathogens in Freshwater Amphipods (*Gammarus lacustris*) from Kazakhstan: Implications for Aquaculture Biosecurity

Contents

Supplementary Table S1. Comprehensive list of viral taxa identified in Gammarus lacustris samples collected from six freshwater ecosystems across Kazakhstan. 1

Supplementary Table S1. Comprehensive list of viral taxa identified in Gammarus lacustris samples collected from six freshwater ecosystems across Kazakhstan.

Sample ID	Species	Genus	Family	Contig count	Contig length[a], (nt)	Identity [b], %	Host type
Gammarus AKM	*Picornaviridae sp.*	unknown	*Picornaviridae*	391	9520-200	78.65-99.29	vertebrates
Gammarus AKM	*Dicistroviridae SC2803*	unknown	*Dicistroviridae*	24	1746-217	91.06-96.66	invertebrates
Gammarus AKM	*Hammarskog picorna-like virus*	unknown	*Picornavirus*	6	3189-571	97.42-99.41	invertebrates
Gammarus AKM	*Polycipiviridae sp.*	unknown	*Polycipiviridae*	4	1337-312	87.63-93.02	invertebrates
Gammarus AKM	*Marnaviridae sp.*	unknown	*Marnaviridae*	1	1090	87.41	algae, marine
Gammarus AKM	*Nodaviridae sp.*	unknown	*Nodaviridae*	3	756-347	99.09-99.18	invertebrates, vertebrates
Gammarus AKM	*Big Sioux River virus*	unknown	*Dicistroviridae*	1	678	96.49	invertebrates
Gammarus AKM	*Aphis gossypii virus*	*Cripavirus*	*Dicistroviridae*	2	486-318	93.26-97.02	invertebrates
Gammarus AKM	*Cripavirus mortiferum*	*Cripavirus*	*Dicistroviridae*	2	418-380	95.93-98.42	invertebrates
Gammarus AKM	*Aedes vexans densovirus*	unknown	*Parvoviridae*	3	640-297	99.39-100	invertebrates, vertebrates
Gammarus AKM	*Fish parvo-like virus*	unknown	*Parvoviridae*	1	372	98.67	invertebrates, vertebrates
Gammarus AKM	*Sichuan tick circovirus 3*	*Circovirus*	*Circoviridae*	2	326-296	94.08	invertebrates, vertebrates

Gammarus KST	*Picornaviridae sp.*	unknown	*Picornaviridae*	241	10007-200	82.6-99.2	vertebrates
Gammarus KST	*Marnaviridae sp.*	unknown	*Marnaviridae*	2	3552-836	77.23-93.9	algae, marine
Gammarus KST	*Hammarskog picorna-like virus*	unknown	*Picornavirus*	3	1184-353	98.2-98.66	invertebrates
Gammarus KST	*Hubei sediment bastro-like virus*	unknown	*Astroviridae*	1	715	85.85	vertebrates
Gammarus KST	*Cripavirus mortiferum*	*Cripavirus*	*Dicistroviridae*	1	1186	95.16	invertebrates
Gammarus KST	*Dicistroviridae SC2803*	unknown	*Dicistroviridae*	12	1636-205	91.95-94.96	invertebrates
Gammarus KST	*Aedes vexans densovirus*	unknown	*Parvoviridae*	2	364-296	100	invertebrates, vertebrates
Gammarus KST	*Fish parvo-like virus*	unknown	*Parvoviridae*	1	325	98.74	invertebrates, vertebrates
Gammarus KST	*Gemycircularvirus derva1*	*Gemycircularvirus*	*Genomoviridae*	1	269	100	environmental
Gammarus PVL	*Fish parvo-like virus*	unknown	*Parvoviridae*	1	282	96.45	invertebrates, vertebrates
Gammarus PVL	*Sichuan tick circovirus 4*	*Circovirus*	*Circoviridae*	1	316	99.43	invertebrates, vertebrates
Gammarus PVL	*Gemycircularvirus derva1*	*Gemycircularvirus*	*Genomoviridae*	2	310-276	100	environmental
Gammarus PVL	*Brine shrimp reovirus 1*	unclassified *Reovirales*	*Reoviruses*	3	578-321	96.9-98.27	invertebrates
Gammarus NKZ	*Cripavirus mortiferum*	*Cripavirus*	*Dicistroviridae*	1	1775	96.16	invertebrates
Gammarus NKZ	*Big Sioux River virus*	unknown	*Dicistroviridae*	1	391	92.77	invertebrates
Gammarus NKZ	*Fish parvo-like virus*	unknown	*Parvoviridae*	7	374-271	98.29-100	invertebrates, vertebrates
Gammarus NKZ	*Flumine parvovirus 20*	unknown	*Parvoviridae*	4	317-278	95.92-98.4	invertebrates, vertebrates
Gammarus NKZ	*Gemycircularvirus derva1*	*Gemycircularvirus*	*Genomoviridae*	1	299	100	environmental
Gammarus NKZ	*Sichuan tick circovirus 3*	*Circovirus*	*Circoviridae*	1	333	100	invertebrates, vertebrates
Gammarus TRK	*Polycipiviridae sp.*	unknown	*Polycipiviridae*	26	5586-200	77.15-97.92	invertebrates
Gammarus TRK	*Violaceae triatovirus 1*	*Triatovirus*	*Dicistroviridae*	1	313	92.38	invertebrates
Gammarus TRK	*Cripavirus mortiferum*	*Cripavirus*	*Dicistroviridae*	1	319	98.32	invertebrates
Gammarus TRK	*Genomoviridae sp.*	unknown	*Genomoviridae*	1	2417	87.02	environmental

Gammarus TRK	*Fish parvo-like virus*	unknown	*Parvoviridae*	1	317	100	invertebrates, vertebrates
Gammarus TRK	*Sichuan tick circovirus 3*	*Circovirus*	*Circoviridae*	1	275	99.17	invertebrates, vertebrates
Gammarus EKZ	*Picornaviridae sp.*	unknown	*Picornaviridae*	13	3559-200	82.4-95.62	vertebrates
Gammarus EKZ	*Zhejiang sediment noda-like virus 1*	unknown	*Nodaviridae*	1	1601	82.59	invertebrates, vertebrates
Gammarus EKZ	*Marnaviridae sp.*	unknown	*Marnaviridae*	2	632-453	99.38-99.57	algae, marine
Gammarus EKZ	*Fish parvo-like virus*	unknown	*Parvoviridae*	2	362-282	93.81-96.87	invertebrates, vertebrates
Gammarus EKZ	*F. montifringilla ambidensovirus*	*Ambidensovirus*	*Parvoviridae*	1	397	94.88	invertebrates, vertebrates

a Only contigs >200 nt in length were retained for downstream characterization. **b** Sequence identity values were determined by BLASTn comparison against viral reference sequences from the NCBI RefSeq database (E-value $<1 \times 10^{-5}$).

Supplementary Table S2. Reference-based genetic variation analysis of dominant picorna-like viruses.

Sample ID	Region	Closest reference sequence	GenBank accession	Reference length (nt)	Consensus length (nt)	Genome coverage (%)	Mean depth (×)	Number of nucleotide variants	Notes
Gammarus AKM	Akmola (Lake Shnet)	Picornaviridae sp.	ON162258.1	8341	8340	97.93	16052	538	Near-complete consensus genome generated using reference-based mapping
Gammarus KST	Kostanay (Lake Zhamanzharkol)	Picornaviridae sp.	ON162258.1	8341	8341	97.63	5176	476	Near-complete consensus genome generated using reference-based mapping
Gammarus EKZ	East Kazakhstan (Lake Belkol)	Picorna-like virus	OQ722329.1	8576	8576	ND	ND	9	Analyzed separately due to closer similarity to an alternative picorna-like reference lineage

Abbreviations: nt, nucleotide; ND, not determined.
